# Supplementary figures and images for: Clinicopathological findings and imaging features of intraductal papillary neoplasms in bile ducts
Source: PeerJ. 2020 Sep 30;8:e10040. doi: 10.7717/peerj.10040 (PMC7532777; doi:10.7717/peerj.10040)

**Supplementary material**

**
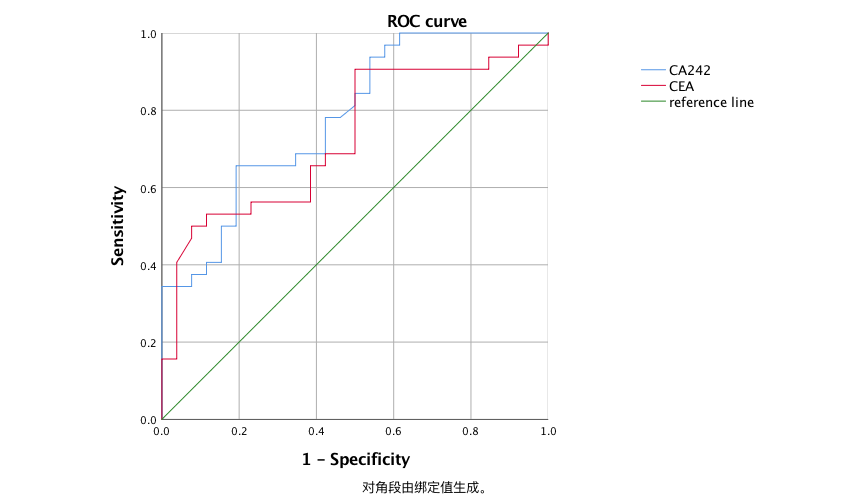
**

The area under the CA242 curve was 0.781.

The area under the CEA curve was 0.728.

Supplement: Supplemental Information 2 [file peerj-08-10040-s002.docx]
